# Glucagon‐like peptide‐1 receptor agonists and kidney outcomes

**DOI:** 10.1111/1753-0407.13609

**Published:** 2024-10-04

**Authors:** Richard J. MacIsaac, Philippa Trevella, Elif I. Ekinci

**Affiliations:** ^1^ Department of Endocrinology & Diabetes St Vincent's Hospital Melbourne Fitzroy Victoria Australia; ^2^ Department of Medicine St Vincent's Hospital Melbourne University of Melbourne Fitzroy Victoria Australia; ^3^ Australian Centre for Accelerating Diabetes Innovations, School of Medicine University of Melbourne Parkville Victoria Australia; ^4^ Department of Endocrinology Austin Health Melbourne Australia; ^5^ Department of Medicine Austin Health, Melbourne Medical School Parkville Victoria Australia

**Keywords:** albuminuria, cardiovascular outcome trials, diabetes, diabetic nephropathy, eGFR, glucagon‐like peptide‐1 receptor agonists, kidney

## Abstract

Glucagon‐like peptide‐1 receptor agonists (GLP‐1RAs) have gained increasing attention for their potential benefits in people with type 2 diabetes mellitus (T2DM) with chronic kidney disease (CKD). Most supportive evidence of a kidney‐protective effect of the GLP‐1RA class of medications has been derived from kidney‐related outcomes reported from cardiovascular outcome trials (CVOTs). GLP‐1RAs have been shown to reduce albuminuria, mitigate cardiovascular risk, and possibly attenuate estimated glomerular filtration rate (eGFR) decline. The kidney‐protective effects of GLP‐1RAs are thought to be attributed to their anti‐inflammatory, antioxidant, and vasodilatory properties. Despite these promising findings, the use of GLP‐RAs has yet to be definitively shown to slow progression to chronic kidney failure in people with T2DM. The Research Study to See How Semaglutide Works Compared to Placebo in People With Type 2 Diabetes and Chronic Kidney Disease (FLOW trial) is the first major trial assessing the potential of a GLP‐1RA to slow progression of kidney disease in people with established CKD to clinically important kidney end points. On March 5, 2024, the top line result from FLOW was announced with semaglutide 1.0 mg being reported to reduce the primary end point of the trial by a significant 24% compared with placebo. Here, we summarize the kidney outcomes reported from CVOTs for the GLP‐1RA class of medication and briefly describe kidney outcomes from other major GLP‐1RAs trials. We also discuss a potential role of the dual GLP‐1/glucose‐dependent insulinotropic polypeptide (GIP) receptor agonist, tirzepatide, as a kidney‐protective agent.

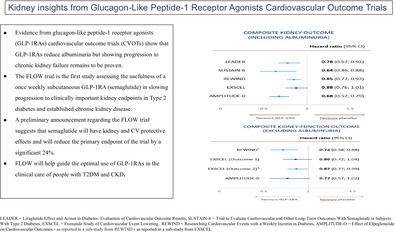

## Graphical abstract

Highlights

- Evidence from GLP‐1RAs CVOTs show that GLP‐1RAs reduce albuminuria but slowing of progression to chronic kidney failure remains to be proven.
- The FLOW trial is the first study assessing the usefulness of a once‐weekly subcutaneous GLP‐1RA (semaglutide) in slowing progression to clinically important kidney end points and protection form cardiovascular disease people with T2DM and established CKD.
- FLOW will help guide the optimal use of GLP‐1RAs in the clinical care of people with T2DM and CKD.

## 1 INTRODUCTION

In addition to the existing requirement for registration studies demonstrating improvements in glycemic control, in 2008, the US Food and Drug Administration (FDA) mandated that the assessment of all new type 2 diabetes (T2D) therapies should include long‐term cardiovascular (CV) outcome trials (CVOTs).^1^ Subsequent CVOTs have demonstrated that some classes of glucose‐lowering therapies, including the sodium glucose cotransporter‐2 (SGLT2) inhibitors and glucagon‐like peptide‐1 receptor agonists (GLP‐1RAs), are not only CV safe but also offer CV protection.^2^ CVOTs of individual medications belonging to the various classes of glucose‐lowering agents that have needed to be performed to comply with the above regulations and have yielded some differences in CV and kidney outcomes as discussed below.

Promising kidney outcome results from these CVOTs paved the way for the designated kidney outcome trials for the SGLT2 inhibitor class of drugs.^3^ These trials have recently established a place for SGLT2 inhibitors as a “pillar” of kidney‐protective pharmacotherapy in people with diabetes and without diabetes.^4^ Multiple CVOTs involving GLP‐1RAs have also been performed over the last 10 years.^5^, ^6^, ^7^, ^8^, ^9^, ^10^, ^11^, ^12^ Some of these trials have shown that GLP‐1RAs can reduce CV events in people with and without diabetes and with and without established CV disease.^3^, ^13^ These trials have also shown that this class of medication reduces albuminuria and most likely slows down kidney function loss. The potential kidney‐protective effects of GLP‐1RAs are thought to be attributed to their anti‐inflammatory, antioxidant, and vasodilator properties.^14^ Recently, RAGE (the receptor for advanced glycation end‐products) has been implicated as a key component of the glucose‐independent kidney‐protective pathways mediated by the actions of GLP‐1.^15^ However, GLP‐1RAs are also known to improve glycemia, blood pressure, and induce weight loss, which could also contribute to their positive effects on kidney health.^16^, ^17^

Despite these promising findings, GLP‐RAs are yet to be definitively shown to slow progression to chronic kidney failure in people with type 2 diabetes mellitus (T2DM). It should be appreciated that GLP‐1RA CVOTs performed to date have involved participants at relatively low risk for progressive kidney function loss. The Research Study to See How Semaglutide Works Compared to Placebo in People With Type 2 Diabetes and Chronic Kidney Disease (FLOW trial, NCT03819153)^18^ was stopped in October 2023 because of efficacy.^19^ On March 5, 2024, the top line result from FLOW was announced with semaglutide 1.0 mg being reported to reduce the primary end point of the trial by a significant 24% compared with placebo.^20^ This is the first major trial assessing the efficacy of a GLP‐1RA to slow the progression of diabetic kidney disease.^18^ Here, we summarize the kidney outcomes reported from GLP‐1RA CVOTs and briefly describe kidney outcomes from other major GLP‐1RA trials to set the scene for the full publication of the FLOW trial results. We also discuss a potential role of the dual GLP‐1/glucose‐dependent insulinotropic polypeptide (GIP) receptor agonist, tirzepatide, as a kidney‐protective agent.

## 2 GLUCAGON‐LIKE PEPTIDE‐1 RECEPTOR AGONIST CARDIOVASCULAR OUTCOME TRIALS

**FIGURE 1:**
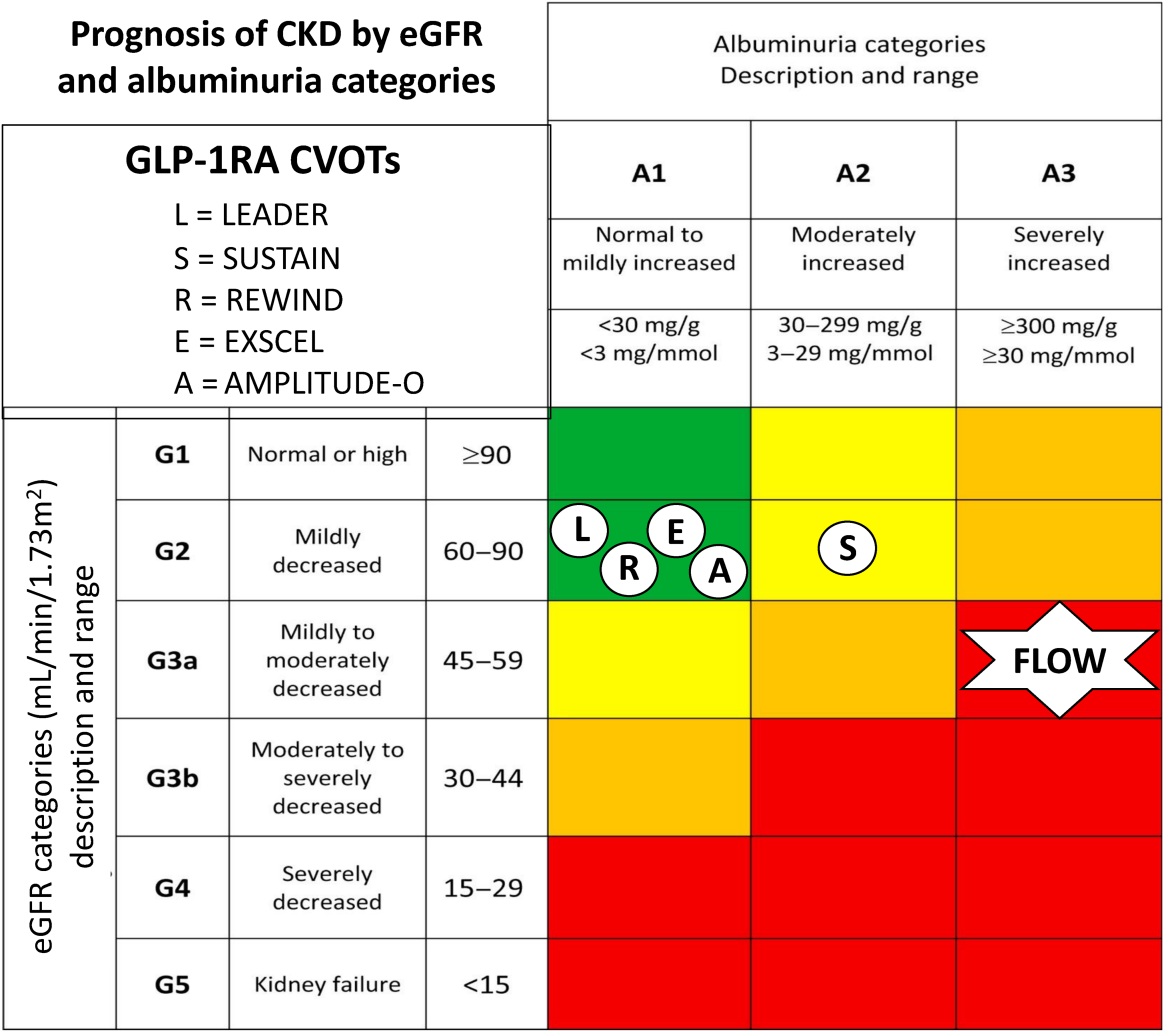
Baseline chronic kidney disease (CKD) staging for the five glucagon‐like peptide‐1 receptor agonists (GLP‐1RAs) cardiovascular outcome trials (CVOTs) (as described in Table 1) compared with the Research Study to See How Semaglutide Works Compared to Placebo in People With Type 2 Diabetes and Chronic Kidney Disease (FLOW) trial.^6^, ^7^, ^8^, ^10^, ^12^, ^18^ Green: low risk (if no other markers of kidney disease, no CKD); yellow: moderately increased risk; orange: high risk; red: very high risk. AMPLITUDE‐O, Effect of Efpeglenatide on Cardiovascular Outcomes; eGFR, estimated glomerular filtration rate; EXSCEL, Exenatide Study of Cardiovascular Event Lowering; LEADER, Liraglutide Effect and Action in Diabetes: Evaluation of Cardiovascular Outcome Results; REWIND, Researching Cardiovascular Events With a Weekly Incretin in Diabetes; SUSTAIN‐6, Trial to Evaluate Cardiovascular and Other Long‐Term Outcomes With Semaglutide in Subjects With Type 2 Diabetes.

The aim of these trials was to assess the CV safety of the GLP‐1RA class of medication on a background of normal clinical care. They have shown that GLP‐1RAs are not only safe from a CV perspective but also have the potential to provide CV protection in the setting of T2DM.^3^, ^16^ GLP‐1RAs also reduce weight in people without diabetes, and recently, the GLP‐1RA semaglutide, at a dose of 2.4 mg per week, reduced CV events in people without diabetes who had preexisting atherosclerotic CV disease and a body mass index (BMI) ≥27 kg/m^2^ in the Semaglutide Effects on Cardiovascular Outcomes in People With Overweight or Obesity (SELECT) trial.^21^ Many of the GLP‐1RA CVOTs in the setting of T2DM have also assessed kidney outcomes as secondary end points. Participants in these trials generally do not have established chronic kidney disease (CKD) and hence kidney‐related outcomes from GLP‐1RA CVOTs, which have also not been the primary end point of these studies, need to be interpreted with caution (Figure 1). The characteristics of participants in GLP‐1RA CVOTs performed in people with T2DM to date are summarized in Table 1.

**TABLE 1:** Characteristics of the major glucagon‐like peptide‐1 receptor agonists (GLP‐1RAs) cardiovascular outcome trials (CVOTs) that have reported on a combined kidney outcome.

CVOT	Drug	Duration	Major inclusion criteria	Kidney status at baseline
LEADER	Liraglutide	3.8 years	T2DM with HbA1c ≥7.0% and age ≥ 50 years with ≥1 cardiovascular comorbidity, or age ≥ 60 years with ≥1 cardiovascular risk factor	eGFR: 80 mL/min/1.73 m^2^ UACR:21 mg/g
SUSTAIN‐6	Semaglutide (subcutaneous)	104 weeks	T2DM with HbA1c ≥ 7% and age ≥ 50 years with established cardiovascular disease or chronic kidney disease of Stage 3 or higher, or age ≥ 60 years with ≥1 cardiovascular risk factor	eGFR: 80 mL/min/1.73 m^2^ UACR: 37 mg/g
EXSCEL	Exenatide	3.2 years	T2DM with HbA1c 6.5%–10% and eGFR ≥ 30; 70% had previous cardiovascular event, and 30% had no previous cardiovascular event	eGFR: 77 mL/min/1.73 m^2^ UACR: 23 mg/g
REWIND	Dulaglutide	5.4 years	T2DM, age ≥ 50 years with a previous cardiovascular event or cardiovascular risk factors	eGFR: 75 mL/min/1.73 m^2^ UACR: 16 mg/g
AMPLITUDE‐O	Efpeglenatide	1.8 years	T2DM and either history of CV disease or current kidney disease (eGFR, 25–59.9 mL/min/1.73 m^2^) plus additional CV risk factor	eGFR: 72 mL/min/1.73 m^2^ UACR: 28 mg/g

Abbreviations: AMPLITUDE‐O, Effect of Efpeglenatide on Cardiovascular Outcomes^12^; CV, cardiovascular; eGFR, estimated glomerular filtration rate; EXSCEL, Exenatide Study of Cardiovascular Event Lowering^8^; LEADER, Liraglutide Effect and Action in Diabetes: Evaluation of Cardiovascular Outcome Results^6^; REWIND, Researching Cardiovascular Events With a Weekly Incretin in Diabetes^10^; SUSTAIN‐6, Trial to Evaluate Cardiovascular and Other Long‐Term Outcomes With Semaglutide in Subjects With Type 2 Diabetes^7^; T2DM, type 2 diabetes; UACR, urinary albumin to creatinine ratio.

## 3 STRENGHTS, WEAKNESSES AND INSIGHTS FROM GLP‐1RAs CVOTS

Although CVOTs have showed that treatment with GLP‐1RAs is associated with significant CV and kidney benefits, differences in outcomes between trials have been reported and the representation of trial participants of people with T2DM seen in everyday clinical practice has been called into question.

The strength of CVOTs derives from their expert and meticulous design of randomized, placebo‐controlled trials on the background of existing established therapies. Generally, GLP‐1RAs CVOTs have enrolled participants with T2DM aged 60–66 years old, who were mainly male (54%–69% of participants), with suboptimal glycemic control (HbA1c range, 7.3%–8.9%) and established CV disease who have been followed‐up for a mean of 1.6–5.4 years,^3^ also please see Table 1 for the inclusion criteria for these trials. Of note, the REWIND (Researching Cardiovascular Events With a Weekly Incretin in Diabetes) trial (dulaglutide) had a slightly different design to the other GLP‐1RA CVOTs as only 31% of trial participants had established CV disease, a factor that most likely accounts for its long follow‐up period of 5.4 years.^10^ In this CVOT, the use of dulaglutide was found to be associated with a significant 12% reduction in major CV events (hazard ratio [HR]: 0.88, 95% confidence interval [CI]: 0.79–0.99). Other differences between trials include the fact the two of the CVOTs (EXSCEL [Exenatide Study of Cardiovascular Event Lowering]‐exenatide and AMPLITUDE‐O [Effect of Efpeglenatide on Cardiovascular Outcomes]‐efpeglanatide) involved the use of a GLP‐1RAs, which were not based on the human GLP‐1 sequence.^8^, ^12^ It should also be noted that albiglutide based on the human GLP‐1 structure, which was used in the HARMONY (Effect of Albiglutide, When Added to Standard Blood Glucose Lowering Therapies, on Major Cardiovascular Events in Subjects With Type 2 Diabetes Mellitus) CVOT, has been discontinued and is no longer available in clinical practice.^9^ Although it is difficult to make direct comparisons between trials because of the differences in trial design, five out of seven trials involving the subcutaneous administration of a GLP‐1 have shown significant reductions of major CV events compared with placebo treatment on top of the normal standard of care (range of reduction, 12%–27%). The reduction of major CV events in the other two trials, EXSCEL and ELIXA‐lixisenatide, ranged between 9% and 0%. The failure of lixisenatide to show a CV benefit may be the result of the trial only enrolling patients who had experienced a very recent acute coronary event and the once daily administration of lixisenatide during the trial, which is considered to be a very short acting GLP‐1RA.^5^ The EXSCEL trial also experienced a very high discontinuation rate of the active medication, which may explain its neutral effect. Most other CVOTs have involved the use of long‐acting GLP‐1RAs administered once weekly and have only enrolled participants after a period of stabilization following an acute CV event. The LEADER (Liraglutide Effect and Action in Diabetes: Evaluation of Cardiovascular Outcome Results) trial enrolled similar participants after a period of stabilization from an acute vascular event but tested the effects of a daily injection of liraglutide. This CVOT which followed‐up 9340 participants (81% with established CV disease) for a mean of 3.8 years is the only trial so far to clearly demonstrate a benefit of GLP‐1RAs in terms of reducing the risk of both fatal and nonfatal myocardial infarction (HR: 0.86, 95% CI: 0.73–1.00) and CV death (HR: 0.78, 95% CI: 0.66–0.93).^6^ In addition, the results of the AMPLITUDE‐O trial involving the use of an exendin‐based GLP‐1RA, which showed a 27% decrease in major CV events (HR: 0.74, 95% CI: 0.58–0.92), suggest that despite the results of EXSCEL, GLP‐1RAs based on the nonhuman exenatide structure may have similar CV benefits to those based on human GLP‐1 structure.

One of the weaknesses of GLP‐1RAs CVOTs is that people with T2DM who are eligible for GLP‐1RAs therapy, according to current guidelines, differ from CVOT trail participants in terms of their demographic, clinical, CV, and kidney risk factors, with the prevalence of heart failure in these trials only ranging from 9% to 24%.^22^ People with young onset T2DM or elderly people, those with very poor glycemic control, a low prevalence of CV risk factors, and people from diverse ethic and social‐economic backgrounds are usually not represented in the above trials. Because of the rapidly evolving area of new treatments for metabolic–CV–kidney disorders, GLP‐1RAs CVOTs have only included a very small percentage of participants who are also taking SGLTs inhibitors, and none have included participants taking the nonsteroidal mineralocorticoid receptor antagonist finerenone, which has recently been shown to have CV‐kidney‐protective effects in people with T2DM and CKD.^23^ Furthermore, as mentioned above, and of particular relevance to this review, most participants in GLP‐1RA CVOTs had well‐preserved kidney function with trials usually excluding participants with estimated glomerular filtration rate (eGFR) <30 mL/min/1.73 m^2^. For the five GLP‐1RA CVOTs that reported on a combined kidney outcome, baseline eGFR values ranged between 72 and 82 mL/min/1.73 m^2^ and ACR values ranged between 16 and 18 mg/g (Table 1).

Reassuringly, a recent analysis of studies that reported on real‐word effects of GLP‐1RAs on CV outcomes in people with T2DM, who were generally at lower CV risk that the above CVOTs, appears to also support a CV protective effect of GLP‐1RAs. The combined results of seven real world studies, excluding SGLT2 inhibitor studies, suggested that GLP‐1RAs were associated with a 30% reduction in major CV events compared with other glucose lower medications (HR: 0.7, 95% CI: 0.58–0.84).^24^

## 4 COMPOSITE KIDNEY OUTCOME END POINTS FROM GLP‐1RA CVOTs

**FIGURE 2:**
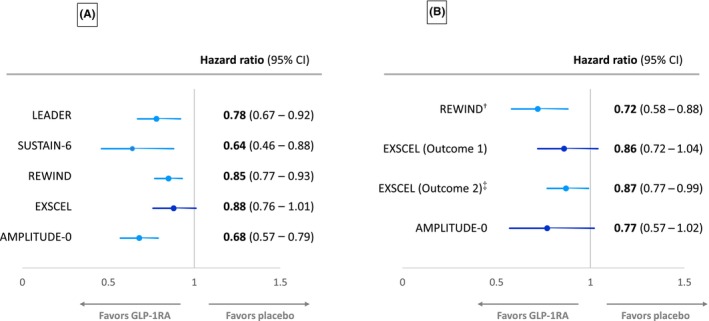
Forest plots of (A) the composite primary kidney outcome (including albuminuria) and (B) the composite kidney function outcome (excluding albuminuria) in major glucagon‐like peptide‐1 receptor agonists (GLP‐1RAs) cardiovascular outcome trials (CVOTs) described in Table 1. Definitions for composite kidney outcomes are given in Table 2.^6^, ^7^, ^8^, ^10^, ^12^, ^23^, ^25^ AMPLITUDE‐O, Effect of Efpeglenatide on Cardiovascular Outcomes; CI, confidence interval; EXSCEL, Exenatide Study of Cardiovascular Event Lowering; LEADER, Liraglutide Effect and Action in Diabetes: Evaluation of Cardiovascular Outcome Results; REWIND, Researching Cardiovascular Events With a Weekly Incretin in Diabetes; SUSTAIN‐6, Trial to Evaluate Cardiovascular and Other Long‐Term Outcomes With Semaglutide in Subjects With Type 2 Diabetes.

The definition of the exploratory primary composite kidney outcome end points has varied between CVOTs for GLP‐1RAs (Table 2A). These definitions have included the onset of macroalbuminuria, and some or all the following outcomes: doubling of serum creatinine or worsening of eGFR by ≥30% or 40%, persistent need for kidney replacement therapy, and death due to kidney disease. Outcomes for the primary composite end point from the various GLP‐1RA CVOTs are summarized in Figure 2A. Kidney function composite end points (excluding albuminuria) for the above trials, when reported, are also summarized in Table 2B and outcomes in Figure 2B.

**TABLE 2:** Definitions for composite kidney end points from cardiovascular outcome trials referred to in Table 1.

Trial	(A) Composite primary kidney outcome definition (including albuminuria)
LEADER (2016)	New‐onset persistent macroalbuminuria, persistent doubling of the serum creatinine level and eGFR of ≤45 mL/min/1.73 m^2^, KRT, or death due to renal disease
SUSTAIN‐6 (2016)	New‐onset persistent macroalbuminuria, persistent doubling of serum creatinine level and creatinine clearance <45 mL/min/1.73 m^2^, the need for continuous KRT (in the absence of an acute reversible cause), or death due to renal disease.
REWIND (2019)	New macroalbuminuria, sustained ≥30% decline in eGFR (based on two consecutive eGFR concentrations), or new chronic KRT comprising dialysis or renal transplantation.
EXSCEL (2017)	Incident macroalbuminuria plus an increase in the UACR of ≥30% from baseline, a sustained decrease in the eGFR of ≥40% for ≥30 days, KRT for ≥90 days, or a sustained eGFR of <15 mL/min/1.73 m^2^ for ≥30 days.
AMPLITUDE‐O (2021)	Incident macroalbuminuria (UACR >300 mg/g) plus an increase in the UACR of at least 30% from baseline, a sustained decrease in eGFR of ≥40% for 30 days or more, KRT for 90 days or more, or a sustained eGFR of <15 mL/min/1.73 m^2^ for 30 days or more.

Trial	(B) Composite kidney function outcome definition (excluding albuminuria)
LEADER (2016)	NR
SUSTAIN‐6 (2016)	NR
REWIND (2019)	eGFR reduction ≥40% from baseline at two or more consecutive visits or ESKD (kidney replacement therapy, kidney transplant, or adverse events reported as ESKD) or kidney disease‐related death.^a^
EXSCEL (2017)	Outcome 1: Sustained (two or more consecutive measurements) eGFR reduction of ≥40% or ESKD (chronic dialysis or kidney transplantation). Outcome 2: Sustained eGFR reduction of ≥30% or ESKD (chronic dialysis or kidney transplantation).^b^
AMPLITUDE‐O (2021)	eGFR reduction of ≥40% for ≥30 days, end‐stage kidney disease (defined as dialysis for ≥90 days, kidney transplantation, or an eGFR of <15 mL/min/1.73 m^2^ for ≥30 days), or death from any cause.

Abbreviations: AMPLITUDE‐O, Effect of Efpeglenatide on Cardiovascular Outcomes^12^; eGFR, estimated glomerular filtration rate; ESKD, end‐stage kidney disease; EXSCEL, Exenatide Study of Cardiovascular Event Lowering^8^; LEADER, Liraglutide Effect and Action in Diabetes: Evaluation of Cardiovascular Outcome Results^6^; NR, not reported; REWIND, Researching Cardiovascular Events With a Weekly Incretin in Diabetes^10^; SUSTAIN‐6, Trial to Evaluate Cardiovascular and Other Long‐Term Outcomes With Semaglutide in Subjects With Type 2 Diabetes^7^; UACR, urinary albumin to creatinine ratio.

^a^ As reported in a substudy from REWIND.^23^

^b^ As reported in a substudy from EXSCEL.^25^

The LEADER trial, which compared the GLP‐1RA liraglutide with placebo in patients with T2DM and high CV risk, was the first major study to specifically report on kidney outcomes for a GLP‐1RA.^6^ This analysis assessed time to the development of a primary composite kidney outcome comprising the development of new‐onset persistent macroalbuminuria, doubling of serum creatinine, need for kidney replacement therapy and kidney death in the 9340 trial participants randomized to liraglutide versus placebo treatment over 3.8 years of follow‐up. In this study, the mean eGFR was 80 mL/min/1.73 m^2^ with only 23% of participants having an eGFR <59 mL/min/1.73 m^2^. At randomization, 26.3% and 10.5% of participants had microalbuminuria and macroalbuminuria, respectively. The composite kidney outcome occurred in significantly fewer patients taking liraglutide compared with placebo (5.7% vs. 7.2%, respectively, HR: 0.78, 95% CI: 0.67–0.92, *p* = 0.003).^6^ However, this finding was primarily driven by a reduction in albuminuria, as there was no significant difference in the rate of doubling of serum creatinine or need for kidney replacement therapy between liraglutide‐ and placebo‐treated participants.^25^ Despite the above, a subsequent substudy from LEADER reported an attenuation in eGFR decline for liraglutide‐treated participants who had a baseline eGFR between 30 and 59 mL/min/1.73 m^2^, as discussed later.^25^

A significant reduction in a composite kidney outcome has also been reported for four other CVOTs involving GLP‐1RAs. SUSTAIN‐6 (Trial to Evaluate Cardiovascular and Other Long‐Term Outcomes With Semaglutide in Subjects With Type 2 Diabetes) compared subcutaneous weekly semaglutide (0.5–1.0 mg doses) to placebo in 3297 patients with T2DM and high CV risk, with a reduction in the composite kidney outcome (new‐onset macroalbuminuria, doubling of serum creatinine or eGFR <45 mL/min/1.73 m^2^, or the need for kidney replacement therapy) being reported for semaglutide‐ (4%) versus placebo‐ (6%) treated participants over a median follow‐up time of 2.1 years (HR: 0.64, 95% CI: 0.46–0.88).^7^ Baseline eGFR and albuminuria status in this study were like those in the LEADER study and, as per the LEADER study, in SUSTAIN‐6, there was no significant difference in the rate of doubling of serum creatinine or the need for kidney replacement therapy between semaglutide‐ and placebo‐treated participants.^7^

In REWIND, dulaglutide was compared with placebo treatment in 9901 patients with T2DM and increased CV risk.^10^ Dulaglutide (1.5 mg per week) treatment resulted in a reduction in the kidney composite outcome compared with placebo treatment (development of macroalbuminuria, sustained >30% decline in eGFR or chronic kidney replacement therapy), 17% versus 20%, respectively (HR: 0.85, 95% CI: 0.77–0.93).^10^ The absolute rates for kidney outcomes in this study were much greater than those in LEADER or SUSTAIN‐6, despite the baseline characteristics and the primary kidney outcome being similar in the three trials. The mean follow‐up in REWIND was longer compared with the LEADER and SUSTAIN‐6 trials, at a median of 5.4 years, but the reasons for the comparative increase in kidney outcomes remains unexplained.

Despite the above, a post hoc analysis from REWIND has shown that a composite kidney function–related outcome (≥40% decline in eGFR, end‐stage kidney disease [ESKD] or kidney‐related death) was reduced by 28% (HR: 0.72, 95% CI: 0.58–0.88, *p* = 0.002) for dulaglutide versus placebo treatment (Figure 2B).^26^ Furthermore, the above results were similar for participants stratified based on baseline albuminuria or eGFR.^26^

In AMPLITUDE‐O, the composite kidney outcome of incident macroalbuminuria, a sustained decrease in the eGFR of ≥40%, kidney replacement, and a sustained eGFR of <15 mL/min/1.73 m^2^ was significantly reduced by 32% for efpeglenatide‐ (13%) versus placebo (18%)‐treated participants (HR: 0.68, 95% CI: 0.57–0.79, *p* < 0.001).^12^ Of note, unlike the above three CVOTs, participants in AMPLITUDE‐O could have T2DM and either a history of CV disease or current kidney disease (defined as an eGFR rate of 25.0–59.9 mL/min/1.73 m^2^) plus at least one other CV risk factor. Approximately 30% of the participants had established CKD at randomization in this study. Furthermore, a kidney function outcome (that did not include incident macroalbuminuria) was nonsignificantly reduced by 23% for participants randomized to efpeglenatide treatment in the trial (HR: 0.77, 95% CI: 0.57–1.02).^12^ A further recent study from the AMPLITUDE‐O trial has found that the beneficial effects of efpeglenatide on indices of kidney health were independent of kidney risk category at the time of randomization.^27^

**FIGURE 3:**
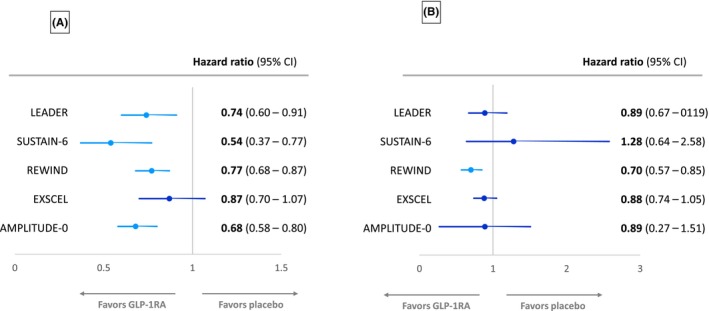
Forest plots of (A) the development of macroalbuminuria and (B) individual measures of worsening of kidney function (estimated glomerular filtration rate [eGFR] reduction of ≥40% or doubling of serum creatinine) in major glucagon‐like peptide‐1 receptor agonists (GLP‐1RAs) cardiovascular outcome trials (CVOTs) described in Table 1.^6^, ^7^, ^8^, ^10^, ^12^ AMPLITUDE‐O, Effect of Efpeglenatide on Cardiovascular Outcomes; CI, confidence interval; EXSCEL, Exenatide Study of Cardiovascular Event Lowering; LEADER, Liraglutide Effect and Action in Diabetes: Evaluation of Cardiovascular Outcome Results; REWIND, Researching Cardiovascular Events With a Weekly Incretin in Diabetes; SUSTAIN‐6, Trial to Evaluate Cardiovascular and Other Long‐Term Outcomes With Semaglutide in Subjects With Type 2 Diabetes.

The EXSCEL trial examined the effects of once‐weekly GLP‐1RA exenatide versus placebo in patients with T2DM with or without preexisting CV or kidney disease.^8^ A composite kidney outcome comprising macroalbuminuria, ≥40% reduction in eGFR, kidney replacement therapy, or death was reduced with exenatide therapy (5.8% exenatide versus 6.5% placebo, respectively), but this reduction just failed to reach statistical significance (HR: 0.88, 95% CI: 0.76–1.01).^28^ Despite this, a kidney function composite end point of ≥30% eGFR decline or ESKD has been reported to be significantly decreased with exenatide treatment compared with placebo (HR: 0.87, 95% CI: 0.77–0.99, *p* = 0.03) in the EXSCEL trial.^29^ Of note, and as shown in Figure 3B, an original report of a kidney function composite end point that contained ≥40% decline in eGFR from the EXSCEL trial suggested that exenatide failed to significantly reduce this end point.^29^

The ELIXA (Evaluation of Lixisenatide in Acute Coronary Syndrome), HARMONY, and PIONEER‐6 (Oral Semaglutide and Cardiovascular Outcomes in Patients With Type 2 Diabetes) trials have not reported on composite kidney end points.^5^, ^9^, ^11^

In a 2021 meta‐analysis involving the LEADER, SUSTAIN‐6, REWIND, EXSCEL, AMPLITUDE‐O, and ELIXA (noting that in ELIXA, only new‐onset macroalbuminuria was included) CVOTs, the composite kidney outcome consisting of development of macroalbuminuria, doubling of serum creatinine or ≥ 40% decline in eGFR, kidney replacement therapy, or death due to kidney disease was reduced by 21% (HR: 0.79, 95% CI: 0.73–087, *p* < 0.0001).^3^ It was estimated that 47 patients needed to be treated over approximately 3 years to avoid this outcome.^3^ In summary, these CVOTs demonstrate an overall reduction in composite kidney outcome events, but this reduction appears to be mainly driven by a reduction in albuminuria.

## 5 ALBUMINURIA IN GLP‐1RAs CVOTs

Albuminuria has been consistently reduced by GLP‐1RAs in CVOTs performed so far, although progression to macroalbuminuria just failed to reach statistical significance in the EXSCEL CVOT (Figure 3A). The LEADER trial found that liraglutide reduced the urinary ACR (UACR) compared with placebo, with a 17% reduction in UACR with liraglutide.^25^ New‐onset macroalbuminuria was reduced by 26% (HR: 0.74, 95% CI: 0.67–0.92, *p* = 0.003) for liraglutide versus placebo.^25^ In the SUSTAIN‐6 trial population, semaglutide significantly reduced UACR from baseline to end of treatment, compared with placebo (treatment ratio 0.74, *p* < 0.001). New‐onset macroalbuminuria was also reduced by 46% (HR: 0.54, 95% CI: 0.37–0.77, *p* = 0.003) for semaglutide versus placebo.^7^, ^30^

In REWIND, dulaglutide reduced new‐onset macroalbuminuria compared with placebo (HR: 0.77, 95% CI: 0.68–0.87).^31^ Overall, although progression to macroalbuminuria just failed to reach significance in EXSCEL, less participants with baseline normoalbuminuria treated with exenatide progressed to develop albuminuria versus placebo treatment during the first 6 months of the trial (3% vs. 4%, *p* = 0.03), but this was mainly accounted for by less transition to microalbuminuria.^29^

In the ELIXA CVOT, lixisenatide reduced UACR compared with placebo in patients with baseline microalbuminuria (−21%, *p* = 0.05) and macroalbuminuria (−39%, *p* = 0.007), but not normoalbuminuria, and reduced the risk for progression to macroalbuminuria (HR: 0.81, 95% CI: 0.66–0.99).^32^ The AMPLITUDE‐O CVOT has also reported that efpeglenatide significantly reduced progression to macroalbuminuria compared with placebo (HR: 0.68, 95% CI: 0.58–0.80).^12^

## 6 KIDNEY FUNCTION LOSS IN GLP‐1RA CVOTs

In addition to the achievement of a kidney function combined end point as discussed above, GLP‐1RAs CVOTs have also reported on separate kidney function outcomes, such as of eGFR change or the achievement of certain eGFR thresholds (usually sustained ≥30 or ≥40% eGFR loss) or doubling of serum creatinine as shown in Figure 3B. In the LEADER trial, there was a small but significant reduction in rate of decline of eGFR over time with liraglutide versus placebo (2% reduction, *p* = 0.01).^25^ As mentioned, subgroup analysis revealed that the significant difference in rate of eGFR decline occurred only in those patients with a baseline eGFR of 30–59 mL/min/1.73 m^2^.^25^ Further post hoc analysis of the LEADER and SUSTAIN‐6 trials has shown that eGFR slope decline was slowed by 0.87 (*p* < 0.0001) and 0.26 mL/min/1.73 m^2^/year (*p* < 0.001) with liraglutide and semaglutide, respectively, with the preservation of eGFR with both GLP‐1RAs also being greatest in participants with baseline eGFR <60 mL/min/1.73 m^2^.^33^ A pooled analysis of the effects of semaglutide and liraglutide revealed a significantly lowered risk of persistent <40% (HR: 0.86, 95% CI: 0.75–0.99, *p* = 0.039) and <50% eGFR decline (HR: 0.80, 95% CI: 0.66–0.97, *p* = 0.023), respectively, versus placebo.^33^ In addition, a combined analysis of semaglutide CVOTs, SUSTAIN‐6 (subcutaneous weekly semaglutide) and PIONEER‐6 (oral daily semaglutide), has also shown that semaglutide therapy can slow annual eGFR slope compared with placebo and again confirmed that this effect was most prominent in participants with a baseline eGFR <60 mL/min/1.73 m^2^.^30^

Post hoc analysis of EXSCEL found that the rate of eGFR decline was reduced in the exenatide group for those patients with a baseline UACR>100 mg/g (treatment effect 0.79 mL/min/1.73 m^2^ per year, *p* = 0.005) and UACR>200 mg/g (treatment effect 1.32 mL/min/1.73 m^2^ per year, *p* = 0.0005), but not in those with lower UACR.^34^ However, in REWIND, the rate of sustained eGFR reduction of ≥30% was nonsignificantly reduced in dulaglutide versus placebo groups (HR: 0.89, 95% CI: 0.78–1.01).^31^ Likewise, the HARMONY study, comparing the GLP1‐RA albiglutide to placebo, found no significant difference in eGFR between groups at 16 months of follow‐up.^9^ The AMPLITUDE‐O trial also found no difference in the change in eGFR over the 1.81 years in efpeglenatide‐ and placebo‐treated participants, −2.37 versus −3.26 mL/min/1.73 m^2^, respectively, with a difference of 0.9 mL/min/1.73 m^2^ (95% CI: 0.3–1.5).^12^

Despite the above, a post hoc analysis from REWIND has shown that the annual decline in eGFR slope was significantly less for participants treated with dulaglutide versus placebo over 5.4 years of the trial's duration (1.37 vs. 1.56 mL/min/1.73 m^2^ per year, *p* < 0.001).^26^ Achievement of the end point of a sustained eGFR decline of 40% was also significantly less for dulaglutide‐ (3.2%) versus placebo‐treated (4.3%) participants (HR = 0.72, 95% CI: 0.58–0.88, *p* = 0.002), unlike the eGFR decline of 30% mentioned above.^26^

In the above‐mentioned meta‐analysis of ELIXA, LEADER, SUSTAIN‐6, REWIND, EXSCEL, and AMPLITUDE‐O CVOTs, the worsening of kidney function outcome defined as either doubling of serum creatinine or ≥40% decline in eGFR, or kidney replacement therapy, or death due to kidney disease was nonsignificantly reduced by 14% (HR: 0.86, 95% CI: 0.72–1.02). However, with the exclusion of the ELIXA CVOT that recruited patients with a recent acute coronary event, unlike other CVOTs, which only included participants with stable CV disease, the HR for the above outcome improved (0.82, 95% CI: 0.69–0.98).^3^ Overall, these data support the reduction of the primary exploratory composite kidney outcomes from the GLP‐1RA CVOTs being mainly being driven by reduction in albuminuria, with some evidence of slowing of kidney function loss. However, slowing of the development of chronic kidney failure with GLP‐1RAs remains to be proven.

## 7 PROGRESSION TO CHRONIC KIDNEY FAILURE

Progression to chronic kidney failure was not reported in all GLP‐1RA CVOTs. In studies reporting on the outcome of progression to ESKD, some have also separately shown this outcome as the need for chronic kidney replacement therapy or the achievement of a sustained eGFR <15 mL/min/1.73 m^2^. In the REWIND study, dulaglutide versus placebo nonsignificantly reduced ESKD (0.6% vs. 0.9%, HR: 0.98, 95% CI: 0.49–1.25), sustained eGFR <15 mL/min/1.73 m^2^ (0.4% vs. 0.5%, HR: 0.69, 95% CI: 0.3–1.21), and kidney replacement therapy (0.3% vs. 0.4%, HR: 0.85, 95% CI: 0.44–1.65), respectively.^31^ Other trials have reported that the need for chronic kidney replacement therapy has been nonsignificantly reduced with various GLP‐1RAs (Table 3). The above outcomes should not be surprising as most of the trial participants in these CVOTs did not have established CKD, so represented a low‐risk population for progression to chronic kidney failure with absolute numbers for this end point being relatively small. For example, in REWIND, 16/4929 (0.3%) of dulaglutide‐ versus 21/4952 (0.4%) placebo‐treated participants progressed to require chronic kidney replacement therapy. In contrast, in the CREDENCE trial that involved the use of an SGLT2 inhibitor in a high‐risk CKD population (eGFR 56 mL/min1.73 m^2^ and UACR 927 mg/g), progression to chronic kidney replacement was significantly reduced in 76/2202 (3.5%) versus 100/2199 (4.6%) trial participants treated with canagliflozin versus placebo, respectively (HR: 0.74, 95% CI: 0.55–1.00).^35^ The analysis of the relative contribution of ESKD development and progression to chronic kidney replacement therapy to the primary end point of the FLOW trial is eagerly awaited.

**TABLE 3:** Proportion of participants progressing to chronic kidney replacement therapy in glucagon‐like peptide‐1 receptor agonists (GLP‐1RAs) cardiovascular outcome trials (CVOTs) as shown in Table 1.^7^, ^22^, ^25^, ^28^

CVOT	Chronic kidney replacement therapy		HR (95% CI)
	GLP‐1RAs (%)	Placebo (%)	
LEADER (liraglutide)	59/4668 (1.2)	64/4672 (1.4)	0.87 (0.61–1.24)
SUSTAIN‐6 (semaglutide)	11/1648 (0.67)	12/1649 (0.73)	0.91 (0.4–0.91)
REWIND (dulaglutide)	16/4949 (0.3)	21/4952 (0.4)	0.75 (0.39–1.44)
EXSCEL (exenatide)	7/7132 (0.098)	7/7137 (0.098)	NR

Abbreviations: CI, confidence interval; EXSCEL, Exenatide Study of Cardiovascular Event Lowering; LEADER, Liraglutide Effect and Action in Diabetes: Evaluation of Cardiovascular Outcome Results; HR, hazard ratio; NR, not reported; REWIND, Researching Cardiovascular Events With a Weekly Incretin in Diabetes; SUSTAIN‐6, Trial to Evaluate Cardiovascular and Other Long‐Term Outcomes With Semaglutide in Subjects With Type 2 Diabetes.

## 8 SUMMARY OF KIDNEY OUTCOMES REPORTED IN OTHER GLP‐1RAs STUDIES

The AWARD‐7 trial compared glycemic control and kidney outcomes for 576 patients with T2DM and moderate to severe CKD treated with the GLP‐1RA dulaglutide versus titrated insulin glargine (U100).^36^ While both agents achieved similar degrees of glycemic control, dulaglutide (1.5 mg dose only) was more efficacious in slowing eGFR decline and reduced the risk of a composite kidney end point comprising reduction of eGFR by ≥40% or persistent kidney replacement therapy versus the insulin group (5.2% vs. 10.8%, *p* = 0.04) over the 52 weeks of the trial.^37^ At least two retrospective cohort studies have also suggested that GLP‐1RAs can lower kidney function loss in comparison to insulin use.^38^, ^39^

## 9 THE DUAL GLP‐1/GIP RECEPTOR AGAINST, TIRZEPATIDE—A KIDNEY‐PROTECTIVE AGENT?

Tirzepatide is a dual GLP‐1 and GIP agonist that also possibly has kidney‐protective benefits. This medication has already been shown to cause unprecedented reductions in HbA1c, clinically significant weight loss, and other metabolic benefits in people with T2DM.^40^ Tirzepatide has consistently been shown to reduce albuminuria in the setting of T2DM. A recent meta‐analysis of 9533 participants in eight randomized controlled trials has shown that tirzepatide produced a significant decrease in albuminuria compared with placebo or insulin treatment (by 32% and 29%, respectively). However, no significant difference in albuminuria reduction was seen compared with GLP‐1RA treatment.^41^

A post hoc analysis of the SURPASS‐4 trial, which compared the effects of tirzepatide versus titrated insulin glargine, showed that tirzepatide slowed the rate of eGFR decline (−1.4 versus −3.6 mL/min/1.73 m^2^ per year) and reduced a composite kidney end point (time to first occurrence of eGFR decline of ≥40% from baseline, ESKD, death from kidney failure, or new‐onset macroalbuminuria) compared with insulin glargine (HR: 0.58, 95% CI: 0.43–0.83), respectively, during a mean treatment duration of 85 weeks.^42^ The reduction in eGFR decline with tirzepatide was more pronounced in trial participants with baseline eGFR <60 mL/min/1.73 m^2^. Similar levels of glycemic control were achieved with both medications, suggesting that the above kidney outcomes seen with tirzepatide were independent of achieved HbA1c levels. Furthermore, as the use of tirzepatide was associated with a reduction of body weight in this study and loss of muscle mass may have influenced creatinine derived measures of GFR, another subsequent substudy of SURPASS‐4 replicated the results using an eGFR derived from cystatin C.^43^

## 10 THE FLOW TRIAL

The FLOW trial, which started in 2019, will be the first GLP‐1RA‐dedicated kidney outcome trial and will assess whether weekly treatment with semaglutide protects against kidney function loss and ultimately slows progression to chronic kidney failure.^18^ This is an international, randomized, double blind, placebo‐controlled trial in which participants (*n* = 3533) have T2DM and established kidney disease (defined as eGFR 50–75 mL/min/1.73 m^2^ and UACR 300–5000 mg/g, or eGFR 25‐50 mL/min/1.73 m^2^ and UACR 100–5000 mg/g).^18^ Participants have been randomized 1:1 to weekly subcutaneous semaglutide (1 mg dose) or placebo in addition to standard of care treatment including RAS inhibition.

The primary end point of the trial, after 3–5 years of exposure, will be time to onset of a composite end point comprising onset of kidney failure (need for ongoing kidney replacement therapy or persistent eGFR <15 mL/min/1.73 m^2^), death from kidney failure or CV causes and persistent ≥50% decrease in eGFR. Secondary end points will include annual rate of change of eGFR, a composite CV end point and time to onset of the individual components of the primary composite end point.^18^ On October 10, 2023, the sponsor of the FLOW trial, Novo Nordisk, announced the decision to stop the trial after an interim analysis suggested superior efficacy with semaglutide treatment.^19^ Subsequently, on March 5, 2024, Novo Nordisk announced that there was a significant 24% reduction in the primary end point for the FLOW trial with semaglutide versus placebo treatment.^20^ Furthermore, the above press release stated that the CKD and CV components of the primary end point contributed to the risk reduction and that many of the secondary end points showed superiority for semaglutide treatment. It was also reported that semaglutide had a well‐tolerated adverse event profile in the FLOW trial that was similar to previous GLP‐1RA trials. We await peer viewed publication of the main analysis from the FLOW trial.

## 11 MECHANISTIC GLP‐1RA STUDIES

The REMODEL study, which is currently in progress, will complement the above outcome trials through its aim to elucidate the mechanism of action of semaglutide on the kidney.^44^ Adults with T2DM and kidney disease (eGFR 40–75 mL/min/1.73 m^2^ and UACR 30–5000 mg/g), who are already stable on RAS inhibition, will be randomized 2:1 to weekly subcutaneous semaglutide 1 mg or placebo in addition to standard of care. Subjects enrolled in this study will have a baseline kidney biopsy and functional magnetic resonance imaging (MRI), followed by repeat MRI at week 4 and repeat biopsy and MRI at week 52.^44^ MRI‐based primary end points will include change in kidney oxygenation, perfusion and inflammation. This study will provide new mechanistic insights into potential hypoxia and inflammation related kidney‐protective mechanisms of GLP‐1RAs.

## 12 POSSIBLE KIDNEY‐PROTECTIVE USE OF GLP‐1RAs POST FLOW

It is interesting to speculate on the positioning of GLP‐1RAs post the publication of the FLOW trial results. Indeed, a recent review has already suggested that GLP‐1RAs could be the fourth pillar of diabetic kidney disease pharmacotherapy along with RAS inhibition, SGLT inhibitors, and finerenone.^4^ Current guidelines including the 2022 Kidney Disease Improving Global Outcomes (KDIGO) guideline recommend the use of GLP‐1RA for those who have not achieved individualized glycemic targets, despite the use of metformin and SGLT2 inhibitor treatment, in people with T2DM and CKD.^45^ The 2024 American Diabetes Association Standards of Care also recommends that in people with T2DM and CKD, a GLP‐1RA should be added to SGLT2 inhibition and metformin if needed to individualize glycemic targets.^46^ Guidelines also emphasize the importance of counseling patients regarding the side‐effect profile of GLP1‐RAs, especially those related to nausea and vomiting. Also, information on the use of GLP‐RAs in the setting of very advanced kidney disease and kidney transplantation is currently very limited.

Overall, there is no definitive evidence of additional kidney benefit with combined GLP‐1RA and SGLT2 inhibitor use, although there is a suggestion of potential benefit from some studies above, and discussed further below.^47^, ^48^ The role and potential additive kidney benefit of GLP‐1RAs in combination with SGLT2 inhibitors is an area of ongoing research interest. Of note, when the FLOW trial commenced, only approximately 15% of participants were on SGLT2 inhibitors, and, as expected, no participant was on finerenone.^18^ This is an important issue as SGLT2 inhibition and finerenone are now accepted as a standard of care, if tolerated, in patients with T2DM and CKD.^4^

Indeed, recent observational evidence has suggested that the combined use of a GLP‐1RA and SGLT2 inhibitor is associated with a lower risk of major CV and kidney adverse events compared with the use of either class of medication alone.^49^ Further evidence supporting the combination GLP‐1RAs, SGLT2i, and finerenone in addition to RAS inhibitor therapy, “the 4‐pillars” of therapy for Diabetic Kidney Disease, has been presented in a recent actuarial analysis of major studies involving the separate use of these medications. This analysis suggested that for patients with T2DM and moderate albuminuria, the estimated treatment effects with SGLT2i, GLP‐1 RA, and finerenone in combination, when added to RAS blockade, compared with conventional therapy could result in a slowing of CKD progression by 33% reduction (HR: 0.67, 95% CI: 0.55–0.80) and produce a 36% reduction in CV death (HR: 0.64, 95% CI: 0.51–0.80). For a 50‐year‐old patient, the combined treatment of the above classes of medications was also suggested to potentially convey gains in freedom from kidney‐related events (5.5 years, [95% CI, 4.0–6.7]) and CV death (2.2 years, [95% CI: 1.1–3.5]) compared with conventional treatment.^50^ Although patients will most likely achieve benefits from the combination of GLP‐1RA, SGLT2i and finerenone therapy, the optimal way to initiate therapy and to subsequently add on additional medications remains to be defined. Of note, the KDIGO guidelines emphasize an individualized approach to treatment with cost being appreciated as a significant barrier to the adoption of guideline recommend therapies for many patients.^45^

Despite the above advances, significant number of individuals with T2DM will progress to ESKD. Novel therapies targeting the molecular pathways involved in the pathogenesis of Diabetic Kidney Disease, including inflammation, oxidative stress, fibrosis, and endothelial dysfunction are already in progress.^51^ Perhaps precision medicine will help to identify patients that will optimally respond to various combinations of the proposed “4‐pillars” of diabetic kidney disease, which possibly in the future will also be the background therapy that the novel therapies mentioned above will be added to.^52^

After the results of the FLOW trial are reflected on, it will also be interesting to see if CKD outcome trials of GLP‐1RAs in people with type 1 diabetes are started. Mechanistic studies such as the Trial of Semaglutide for Diabetic Kidney Disease in Type 1 Diabetes (RT1D) have already been planned (NCT05822609).^53^ A role for GLP‐1RAs in the setting of CKD that is not related to diabetes also remains speculative.

## 13 CONCLUSIONS

CVOTs have demonstrated that GLP‐1RAs reduce albuminuria and may reduce the rate of eGFR decline in people with T2DM. However, protection from progression to chronic kidney failure by GLP1‐RAs has not yet been proven. The FLOW trial is the first study assessing the usefulness of a once‐weekly subcutaneous GLP‐1RA (semaglutide) in slowing progression to clinically important kidney end points in participants with T2DM and established CKD. The FLOW trial will also provide important information on the CV protective effects of semaglutide in the above setting. These results will also have great relevance to the management of people with T2DM and CKD given the well know association between increasing CV risk between increasing albuminuria and/or declining kidney function.^45^

A preliminary announcement from the sponsors of the FLOW trial suggests that semaglutide will have kidney and CV protective effects and will reduce the primary end point of the trial by a significant 24%. We await the full peer reviewed publication of the main results from FLOW to guide the optimal use of GLP‐1RAs in the clinical care of people with T2DM and CKD.

## FUNDING INFORMATION

Open access publishing facilitated by The University of Melbourne, as part of the Wiley–The University of Melbourne agreement via the Council of Australian University Librarians. WOA Institution: The University of Melbourne. Consortia Name: CAUL 2023.

## CONFLICT OF INTEREST STATEMENT

Richard J MacIsaac and Elif I Ekinci have been investigators in multiple clinical trials involving glucagon‐like peptide‐1 receptor agonists. The results of some of these trials are reviewed in this article.
